# Engaging publics in biobanking and genetic research governance - a literature review towards informing practice in India

**DOI:** 10.12688/wellcomeopenres.16558.2

**Published:** 2021-02-16

**Authors:** Prasanna Warrier, Calvin Wai-Loon Ho, Susan Bull, Mario Vaz, Manjulika Vaz

**Affiliations:** 1 Health and Humanities, St John's Research Institute, Bengaluru, Karnataka, 560034, India; 2 Faculty of Law and Centre for Medical Ethics and Law, The University of Hong Kong, Pok Fu Lam, Hong Kong SAR, China; 3 Ethox Centre and Wellcome Centre for Ethics and Humanities, University of Oxford, Oxford, OX3 7LF, UK

**Keywords:** Public, Community, Stakeholder, Engagement, Biobank, Genetic research, Ethics, India

## Abstract

**Background:** There is growing interest in advancing biobanking and genetic research in many countries, including India. Concurrently, more importance is being placed on participatory approaches involving the public and other stakeholders in addressing ethical issues and policymaking as part of a broader governance approach. We analyse the tools, purposes, outcomes and limitations of engaging people towards biobanking and genetic research governance that have been undertaken worldwide, and explore their relevance to India.

**Methods:** Papers to be reviewed were identified through a targeted literature search carried out using ProQuest and PubMed. Retrieved papers were analysed with the Rpackage for Qualitative Data Analysis using inductive coding and thematic analysis, guided by the Framework Method.

**Results:** Empirical studies on public and community engagement in the context of biobanking and or genetic research show a predominance towards the end of the last decade, spanning 2007 to 2019. Numerous strategies—including public meetings, community durbars, focus group discussions, interviews, deliberations, citizen-expert panels and community advisory boards—have been used to facilitate communication, consultation and collaboration with people, at the level of general and specific publics. Engagement allowed researchers to understand how people’s values, opinions and experiences related to the research process; and enabled participants to become partners within the conduct of research.

**Conclusions:** Constructs such as ‘co-production’, ‘engagement of knowledges’, ‘rules of engagement’ and ‘stewardship’ emerge as significant mechanisms that can address the ethical challenges and the governance of biobanking and genetic research in India. Given the inherent diversity of the Indian population and its varying cultural values and beliefs, there is a need to invest time and research funds for engagement as a continuum of participatory activity, involving communication, consultation and collaboration in relation to biobanking and genetic research. Further research into these findings is required to explore their effective employment within India.

## Introduction

Over the last two decades, significant advances in the field of genetic research and precision medicine have been facilitated by biobanking—the large-scale storage and use of human biological material (HBM) and associated data. Additionally, technological innovations allow substantial amounts of DNA information to be analysed, with decreasing errors ^1^. Biobanking and genetic research hold the promise of improvements in human health and clinical outcomes through translational research in pharmacogenomics and personalised medicine. This potential has been recognised in India ^2, 3^, but biobanking and genetic research are also associated with distinct ethical concerns, including issues related to secondary use of stored samples, informed consent, trust, benefit sharing, incidental findings, privacy and confidentiality ^4– 6^. Eliciting public perceptions within India, on biomedical research using stored HBM samples and genome editing has found that there is a lack of confidence in regulatory processes among people, who believe mechanisms such as broad consent are instituted to primarily protect researchers interests ^7, 8^. Public engagement has the potential to build trust, accountability and fair research practices, however it is still at a nascent stage in India ^8, 9^.

The success of biobanking and genetic research requires the participation of a large number of people and public support for the research ^10^. Therefore, to sustain participation and support, it is important to engage the public to understand their concerns about the tenets of biobanking and genetic research, particularly in determining how ethical issues pertaining to the use of HBM and data may be resolved ^10– 13^. The need to engage specific publics—patient groups, research participants, scientists, policymakers and ethnolinguistic groups among other stakeholders—has also become apparent owing to differences in people’s values and beliefs about the inheritance of disease; the sensitivities in the collection, storage and use of HBM and associated data; and, what they view as the ideal conduct of biobanking research and its desired outcomes ^7, 12, 14, 15^. This is more pronounced in research involving the participation of vulnerable populations, including vulnerability emerging from the interplay of race/ethnicity, low socioeconomic status and educational levels ^16– 18^. Thus, engaging people—general and specific publics—particularly through participatory approaches, has emerged as an ethical imperative in policymaking and a means to ensure legitimacy of biobanking and genetic research ^13, 19, 20^. In this regard, participatory approaches are generally composed of methods which highlight subjective differences and give voice to people's varied perspectives and experiences by involving them within the decision making process; rather than deriving conclusions based on statistical methods alone ^13, 21^.

The ‘National Ethical Guidelines for Biomedical and Health Research Involving Human Participants’ of the Indian Council of Medical Research (ICMR) describes procedural requirements and a range of ethical issues pertaining to biobanking and genetic research. They also place an imperative on researchers to include components of community engagement in the research process ^22^. Considering the sociocultural, economic and ethnic diversity in India, interaction with peoples’ values and beliefs—which vary across groups—and the practices involved in biobanking and genetic research can strengthen ethical responses ^7^. However, there are numerous strategies of public and community engagement. Therefore, the purpose of this review is to identify the various approaches of engagement that have been employed worldwide, and explore their possible application in the governance of biobanking and genetic research within India; and to understand why the engagement of general and specific publics, as elaborated below, may bear relevance to sustaining trust and public support for biobanking and genetic research in the Indian context.

## Methods

In line with the purpose of our review, the following questions were formulated to guide our analysis: i) Who were the people engaged? ii) What approaches have been used to engage people across different geographies? iii) What was the purpose of engaging people in biobanking and genetic research? iv) What expectations from biobanking and genetic research governance emerged through engagement? v) How have stakeholder perspectives informed policy and practice in biobanking and genetic research? vi) What are the barriers to conducting engagement?

**Figure 1.**
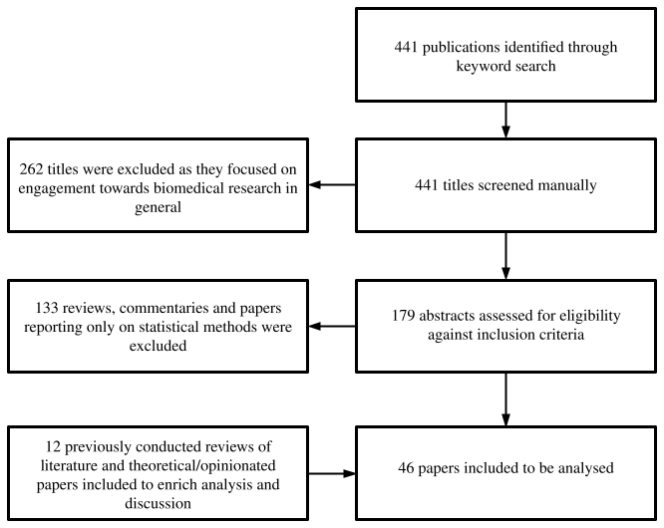
Process of literature search and inclusion in analysis.

A targeted literature search with the keywords, (“Public Engagement” OR “Community Engagement”) AND (“Biobanking” OR “Genetic Research”) was carried out using the ProQuest and PubMed databases, in December 2019 and March 2020 respectively. The initial searches identified 441 publications (386 from ProQuest and 55 from PubMed), which were reviewed against our inclusion criteria. The process of literature search and inclusion is provided in Figure 1.

### Inclusion criteria

Of the 441 publications identified, 179 were relevant to biobanking and genetic research and their abstracts were then subjected to the inclusion criteria which was undertaken manually by one author (PW), then reviewed and verified by another (last author MV). A publication was included in the review if:

1. It was an original paper published in an English peer-reviewed journal;
2. The approach emphasised participation, which gives voice to people's varied perspectives; or
3. The approach adopted mixed research methodologies and emphasised participation or engagement; and
4. The paper reported general or specific publics’ perceptions related to ethical aspects or the governance of biobanking or genetic research.

We focused on the use of participatory approaches as they can be particularly pertinent to the Indian context, where awareness about science and health related issues is low; and an unequal power dynamic exists between the researchers and research participants ^7^. On the basis of this criteria, 133 publications reporting conclusions drawn only from statistical methods to measure people’s opinions, were excluded. 46 papers reporting on empirical findings, published between 2007 and 2019, were included in the analysis. Subsequently, 12 reviews and theoretical/opinion pieces on engagement in biobanking and genetic research ( Table 1) were added to enrich our analysis and discussion, as they reported on findings arising from a number of empirical papers included in this review.

**Table 1.** Reviews and theoretical/opinion pieces included to supplement our analysis.

1.	Avard D, Bucci LM, Burgess M, Kaye J, Heeney C, Cambon-Thomsen A (2009). Public Health Genomics (PHG) and Public Participation: Point to Consider. Journal of Public Deliberation; 5:7
2.	Lemke AA *et al.* (2010). Community engagement in biobanking: Experiences from the eMERGE Network. Genomics, Society and Policy, Vol.6, No.3 pp.50-67
3.	Mello MM and Wolf LE (2010). The Havasupai Indian tribe case—lessons for research involving stored biologic samples. N Engl J Med; 363(3):204-207
4.	O’Doherty K and Hawkins A (2010). Structuring Public Engagement for Effective Input in Policy Development on Human Tissue Biobanking. Public Health Genomics, 13(4): 197–206
5.	Gottweis H, Chen H and Starkbaum J (2011). Biobanks and the phantom public. Human genetics, 130(3), 433–440
6.	Nobile H, Vermeulen E, Thys K, Bergmann MM and Borry P (2013). Why do participants enroll in population biobank studies? A systematic literature review. Expert Rev. Mol. Diagn. 13(1), 35–47
7.	Staunton C and Moodley K (2013). Challenges in biobank governance in Sub-Saharan Africa. BMC Medical Ethics, 14:35
8.	Lemke AA and Harris-Wai JN (2015). Stakeholder engagement in policy development: challenges and opportunities for human genomics. Genetics in Medicine, 17:12
9.	Tindana P *et al.* (2015). Community engagement strategies for genomic studies in Africa: a review of the literature. BMC Medical Ethics, 16:24
10.	Tupasela A, Snell K and Cañada JA (2015). Constructing populations in biobanking. Life Sciences, Society and Policy, 11:5
11.	Domaradzki J and Pawlikowski J (2019). Public Attitudes toward Biobanking of Human Biological Material for Research Purposes: A Literature Review. Int. J. Environ. Res. Public Health, 16:2209
12.	Moodley K and Beyer C (2019). Tygerberg Research Ubuntu-Inspired Community Engagement Model: Integrating Community Engagement into Genomic Biobanking. Biopreservation and Biobanking, 17:6

### Analysis

Text files of the retrieved papers were analysed using the R package for Qualitative Data Analysis (RQDA) software (version 0.3-1, 2018). The Framework Method was employed to guide the analysis of the retrieved papers ^23^. Codes were generated inductively and then subjected to thematic analysis to identify particular patterns emerging from the data. This was constantly iterated between two investigators (PW and last author MV). Emergent themes have been presented in the findings, with respect to our research questions.

## Results

**Figure 2.**
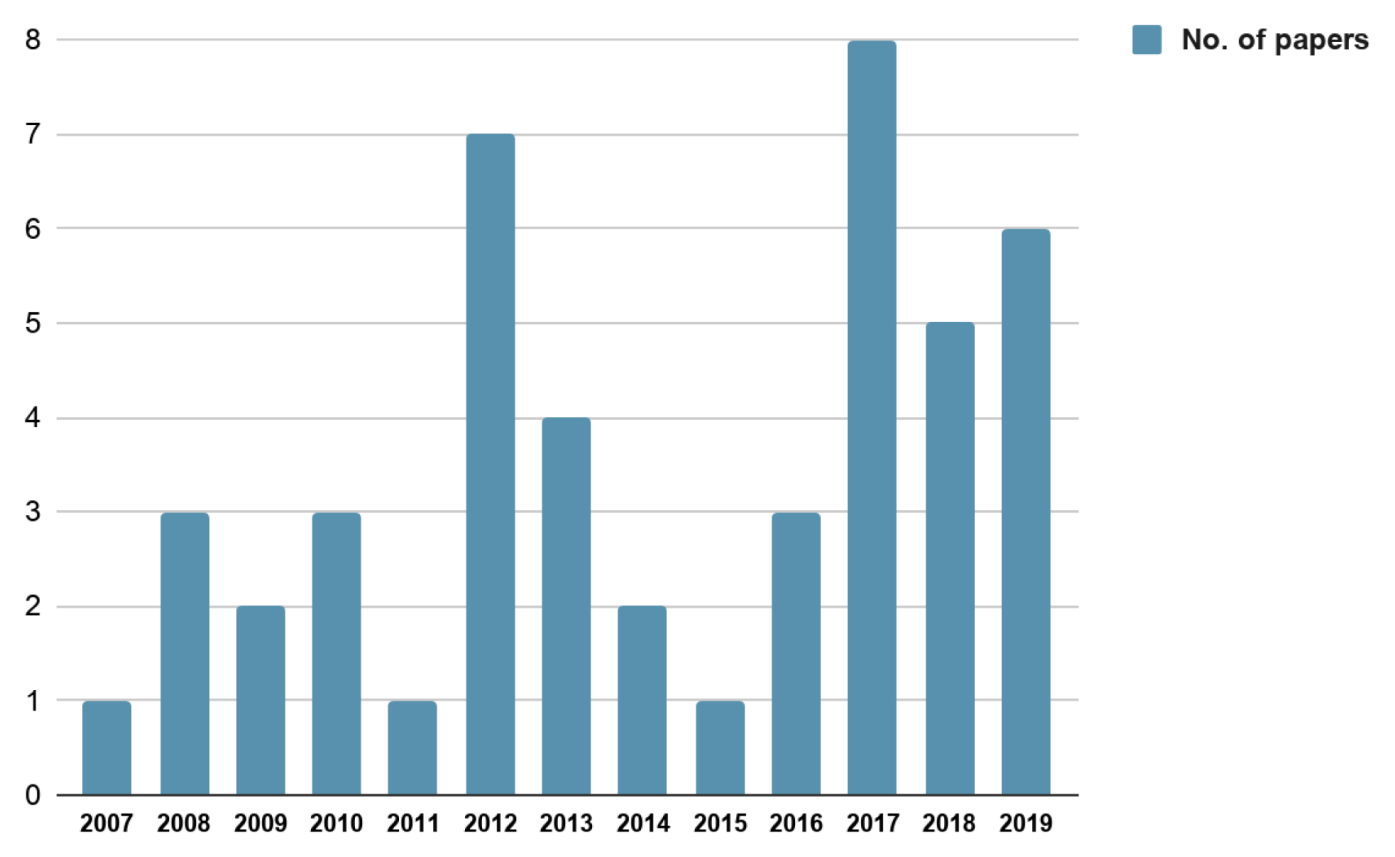
No. of papers published year wise.

The 46 empirical papers included in this analysis reported findings across five continents - Africa (10), Asia (1), Australasia or Oceania (2), Europe (6) and North America (27)—with the timeline of publications spanning from 2007 to 2019 ( Figure 2). 42 papers reported data specific to a single country, while 4 papers reported findings from multiple countries ^24– 27^; and 2 papers reported perceptions from across two continents ^25, 27^. 19 papers reported findings specific to biobanking ^24, 28– 45^, 18 papers reported findings specific to genetic research ^25– 27, 46– 60^ and 9 papers explored the two topics in conjunction ^61– 69^.

### i) Who were the people engaged?

**Figure 3.**
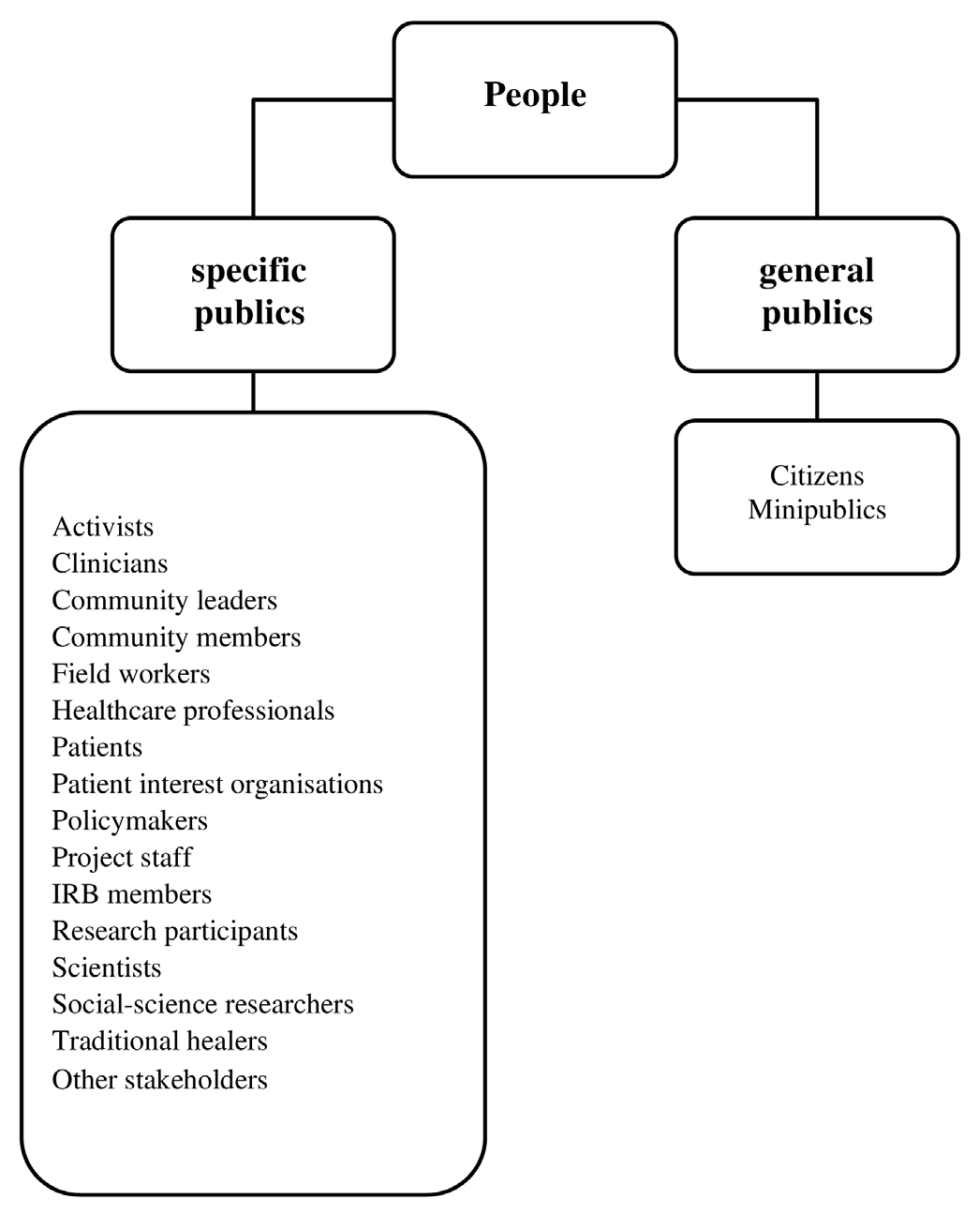
Categories of people engaged across all studies. IRB – Institutional review board.

People engaged belonged to the categories of general and specific publics ^12^ ( Figure 3). Participants at the public level were sometimes referred to as citizens or minipublics, and measures were instituted to ensure diversity in the group, such as including the participations of specific publics ^24, 26, 28– 30, 32, 34, 35, 41, 43, 46, 47, 50, 53, 61– 63^. Specific publics engaged included activists ^24^, auxiliary healthcare professionals ^26, 55, 59, 68^, clinicians ^42, 51, 56^, community members ^31, 33, 40, 42, 45, 54, 55, 57– 60, 64, 67^, community representatives ^26, 31, 54, 59, 64, 69^, field workers ^51^, institutional review board members ^37^, patients ^26, 36, 49, 56, 61, 67^, patient and interest organisations ^25^, policymakers ^26, 56^, project staff ^37, 44, 51^, research participants ^24, 27, 36, 39, 49, 51, 63^, scientists ^37, 38, 42, 44, 50, 51, 56, 65, 68^, social science researchers ^42, 56, 65^ and traditional healers ^26^.

### ii) What approaches have been used to engage people across different geographies?

The majority of the studies reviewed (n=38) used only participatory approaches, while eight papers reported the use of surveys as a tool to statistically measure people’s opinions, in addition to findings that emerged from participatory approaches ^24, 33, 36, 46, 53, 55, 57, 67^.

Focus group discussions ^24, 26, 31– 33, 46– 48, 51, 55– 60, 62, 63^ and interviews ^25– 27, 36– 39, 51, 54, 55, 59, 64, 65, 67, 68^ were the most utilised tools to examine the ethical, legal and social implications (ELSI) of biobanking and genetic research. Deliberative engagement was undertaken to inform the development of policy recommendations for biobanks ^28– 30, 34, 35, 40, 56, 61^. Public meetings and community durbars were used to engage large numbers of people ^48, 51^. Bootcamp translation, workshops and citizen-expert panels honed general and specific publics’ suggestions towards the governance of biobanking and genetic research, while citizens’ reference panels and community advisory boards enabled active participation in the decision making process ^42, 43, 45, 48, 52, 64, 66, 69^. Bootcamp translation and citizen-expert panels aimed to create an interface between experts and publics, while citizen reference panels aimed to represent the views of a particular demographic, in a manner similar to community advisory boards. One study developed the concept of a ‘Genome Diner’ to create an interface between researchers and middle-school students and their parents ^50^. Two studies used online forums; one sought opinions from researchers while the other engaged the general public through social media ^41, 44^.

Studies that included general publics largely made use of random sampling techniques; and in some cases used measures to ensure that participants represented population characteristics ^24, 28– 30, 32, 34, 35, 46, 53, 61^. While engaging specific publics like fieldworkers, scientists, patients and research participants, investigators largely used networks already in place ^24, 27, 39, 40, 44, 49, 51, 65^. A few studies that elicited community perceptions used intermediaries—community representatives and leaders—acting as liaisons, to identify and select participants ^31, 57, 63, 69^.

Multiple models have been devised to describe the level of public involvement in decision making. Drawing from the International Association of Public Participation and Health Canada models of public participation, three broad stages with increasing levels of public involvement in the engagement process have been identified ^13, 21^. In these models, the degree of public participation within biobanking and genetic research has been situated on a continuum that ranges from increasing public awareness on particular aspects of science and technology (communication: low-level engagement); eliciting public opinion on controversial topics towards informing the governance of research (consultation: mid-level engagement); and actively incorporating public input into policy by establishing a dialogue between publics and scientists, policymakers, and other stakeholders (collaboration: high-level engagement) ^13, 21^. The term “community engagement” has been used while including particular stakeholder groups—such as scientists, policymakers, ethnolinguistic groups and other categories of people that are seen as specific publics, who are distinctly different from the broader notion of a general public ^12, 13, 15^. Table 2 places the various tools identified in our review within the context of the participatory continuum. 

**Table 2.** Approaches used to engage people across all studies.

Level of participation	Approaches
*Communication (low engagement)*	1. Bootcamp translation 2. Community durbar 3. Genome diner
*Consultation (moderate engagement)*	1. Focus group discussion 2. Interview 3. Public meeting 4. Online forum 5. Survey 6. Workshop
*Collaboration (high engagement)*	1. Citizen’s reference panel 2. Citizen-Expert panel 3. Community advisory board 4. Deliberation

### iii) What was the purpose of engaging people in biobanking and genetic research?

Engagement of general and specific publics was driven by a variety of purposes. This included, eliciting general and specific publics’ views on the ELSI of biobanking and genetic research and honing their inputs towards the governance of biobanking and genetic research ( Table 3). Engagement has also been used as a mechanism to actively incorporate general and specific publics’ suggestions to govern particular biobanks or genetic studies; these have been differentiated on the basis of whether public or community engagement was reported ( Table 4).

**Table 3.** Studies eliciting people’s perceptions of biobanking and genetic research.

*General* *publics*	Kaufman *et al.*, 2008; Murphy *et al.*, 2008; Lemke *et al.*, 2010; Gaskell *et al.*, 2013; Bombard *et al.*, 2013; Etchegary *et al.*, 2015; Treadwell *et al.*, 2017; Goisauf and Durnová, 2019
*Specific* *publics*	Lemke *et al.*, 2010; Goldenberg *et al.*, 2011; Hiratsuka *et al.*, 2012; Halverson and Ross, 2012; Haukkala *et al.*, 2013; Isler *et al.*, 2013; Haldeman *et al.*, 2014; Ngui *et al.*, 2014; Budin-Ljøsne and Harris, 2016; Longo *et al.*, 2016; Moodley and Singh, 2016; Beaton *et al.*, 2017; Dean *et al.*, 2017; Dixon-Woods *et al.*, 2017; Munung *et al.*, 2017; Tindana *et al.*, 2017; Treadwell *et al.*, 2017; Haring *et al.*, 2018; Ogunrin *et al.*, 2018; Sheikh and Hoeyer, 2018; Staunton *et al.*, 2018; Dennis-Antwi *et al.*, 2019; Dirks *et al.*, 2019; Khabour and Abu-Siniyeh, 2019

**Table 4.** Engagement undertaken by biobanks and genetic studies.

*Public engagement*	Burgess *et al.*, 2008; O'Doherty and Burgess, 2009; Secko *et al.*, 2009; Walmsley, 2010; Duquette *et al.*, 2012; Molster *et al.*, 2012; O'Doherty *et al.*, 2012; Platt *et al.*, 2017; Coors *et al.*, 2018
*Community* *engagement*	Godard *et al.*, 2007; Marsh *et al.*, 2010; O'Daniel *et al.*, 2012; Tindana *et al.*, 2012; Dry *et al.*, 2017; Mosavel *et al.*, 2019; Staunton *et al.*, 2019

In studies conducted across African countries, community engagement was seen as imperative to govern the establishment and conduct of large-scale international collaborative projects using African samples; like those undertaken by the MalariaGEN and H3Africa consortiums ^26, 48, 51, 59, 65, 66^. The majority of studies received international funding from organisations such as the Bill & Melinda Gates Foundation, National Institutes of Health and Wellcome; and focused on understanding how communities’ values and beliefs influenced the conduct of biobanking and genetic research ^26, 48, 51, 58, 59^ and developed methods for engagement needed to address the same ^66, 68, 69^.

Two projects documented researcher views on how to ensure sustainable partnerships between local and international collaborators, owing to a history of exploitation in Africa within the scientific community ^38, 65^. Similarly, a single study from Jordan consulted researchers on their views of ethical concerns accompanying the establishment of a biobank to enhance diabetes research in the country ^44^.

In Australia, the general public was engaged in order to provide recommendations on biobank governance that were adopted as health policy ^34^. Members of the Māori community were engaged in New Zealand to develop a culturally informed model for biobanking projects involving the Māori ^64^.

Within Europe, engagement was conducted to elicit stakeholders’ perspectives on concepts like re-consent, trust and the return of results in biobanking and genetic research ^25, 27, 36, 39, 43^. Prior to these focused studies, a large-scale mixed methods approach was undertaken to document pan-European views regarding the ELSI of creating a biobank network across European countries ^24^.

Engagement towards biobanking and genetic research in Canada echoed several of the purposes discussed above. This included consultations in Quebec to establish the CARTaGENE Project ^46^; public deliberation in British Columbia addressing the ethics, governance, perceptions and expectations of biobanking and genetic research ^28– 30, 35, 53, 61^; and consulting with stakeholders to address the ethical challenges presented by the pharmacogenomics through personalised medicine ^52, 56^.

In the United States, general and specific publics were consulted to understand the factors influencing participation in research for different communities ^31– 33, 37, 41, 45, 49, 55, 57, 60, 62, 63, 67^; expectations with respect to data sharing ^32, 40, 42, 57, 60, 63^ and the return of research results ^33, 40, 47, 60, 62^; and views on the inclusion of children and pregnant women in biobanking and genetic studies ^33, 55, 62^. There was a focus on including the perceptions of minority groups like African-Americans, Hispanics, Alaska Natives and Native Americans, to inform the governance of biobanking and genetic research ^31, 33, 40, 45, 54, 55, 57, 60, 67^. Engagement was also used as a tool to inform geneticists about the public understanding of relevant science ^50^ and translate the science of biobanking into patient-centered language ^42^.

### iv) What expectations from biobanking and genetic research governance emerged through engagement?

Reported findings highlighted the need to increase general awareness about biobanking and genetic research, specifically in minority groups ^32, 40, 42, 44, 45, 49, 50, 52– 55, 59, 63, 70^. The desire of participants to be recognised as partners in the research process, whose opinions and experiences were to be respected, was evident from the views of general and specific publics ^35, 37, 46, 48, 60, 64, 66, 67^. Findings also showed the value of using local knowledge and lay language while communicating about research; and developing innovative ways to communicate the essential characteristics of the research in local languages when exact translations may not be available ^45, 48, 51, 53, 55, 60, 66– 68^.

Ensuring trust was perceived to be imperative in maintaining sustainable relationships between researchers and participants. Trust in the researcher and research institution was a significant factor that guided views on participation and the belief that privacy and confidentiality were protected ^10, 24, 27, 29, 30, 58, 63, 64, 67, 71^. Engaging researchers revealed that the level of trust participants had in them directly influenced the willingness to enrol in research and their continued support ^38, 44^. With respect to the protection of privacy and confidentiality, concerns about genetic discrimination arising from sharing research results were expressed ^29, 32, 42, 43, 53, 56, 57, 63^. Researchers who were engaged noted that maintaining a robust system of de-identification was essential to uphold participants’ trust ^44^; others referred to this process as the ‘myth of anonymity’, owing to the nature of genetic signatures ^38^.

Apart from this, conceptions of trust related to participants’ beliefs that their samples and data were being used for public good, and not to serve commercial interests ^24, 37, 39, 43, 46, 53, 56, 63^. In Scotland, participants suggested having ‘warrants of trust’ to ensure that their participation was towards a common good, proper safeguards were in place and they weren’t exposing themselves to risks by participating ^39^. Trust and hope were sometimes interconnected ^27^. Some people believed that participation would directly benefit them, in the form of free health check-ups or new and more effective therapies; reflective of therapeutic misconception ^27, 48, 51, 71^.

The concept of stewardship—assumed by parents or guardians, community leaders and biobanks—was conceptualised as a “gate-keeping” or custodial role that facilitated the conduct of research in a manner that could uphold people’s specific values and beliefs ^33, 37, 48, 54, 57, 59, 64^. In this context, community representatives have been described as brokers in bridging the gap between researchers and the local community ^70^. Field workers have also been described as cultural brokers who negotiate the trade-off between institutional views on research and people’s expectations from participation, in the process of obtaining informed consent ^48^. These factors highlight the significance of incorporating relational practices to supplement principle-based paradigms of ethics in biobanking and genetic research ^37, 43, 46, 48, 57, 60, 64, 66^.

Employing culturally appropriate strategies was seen as important to guide interaction with participants; especially the vulnerable populations ^55, 67^. Similarly, the need to establish sustainable collaborations in scientific research became clear where knowledge gaps existed among scientists, and a history of exploitation within the research community had been recorded ^38, 65^. These can collectively be termed the ‘rules of engagement’ ^65, 68^. Rules of engagement can be understood as a means of formalising participants’ and researchers’ values, experiences and beliefs within the governance of biobanking and genetic research and its conduct. For example, the Māori believe *Tikanga*, or protocols for research with tissue, should address physical and spiritual components of consent that reflect their idea of sample contribution as *Taonga*—a treasured possession that is gifted to researchers ^64^. Similarly, according to the traditions of one community engaged in Africa, researchers were expected to contact community elders or leaders prior to communicating with community members, to establish their identities and authenticity of their work; following which the representatives informed the community about whether they should participate or not ^58^. African researchers wanted regulations to ensure that local scientists were central to decisions involving the use of African samples and the intellectual property resulting from its research, owing to a history of exploitation in the global scientific community ^38, 65^. Similar concerns were raised by Alaska Native and Havasupai Indian communities, who felt that research and subsequent communications should be approved by the community first, owing to previous experiences with ‘helicopter research’ ^31^; and the misappropriation of their samples in secondary research ^72^.

### v) How have stakeholder perspectives informed policy and practice in biobanking and genetic research?

The incorporation of suggested practices within the conduct of research were largely seen to be the responsibility of researchers and oversight bodies like research ethics committees ^31, 45, 46, 49– 51, 53, 54, 60, 64, 67, 68, 72^. It was apparent, from the papers reviewed, that several positive steps had been taken in the incorporation of stakeholder perspectives.

Engaging publics showed that obtaining broad consent and harmonising ethical frameworks across European countries to integrate biobank networks would be challenging, given people’s diverse experiences and understandings of biobanking ^24^. Research on public engagement undertaken in Austria showed that shifting the focus of engagement from representing different publics to understanding how different ‘knowledges’ interface can be used to inform appropriate guidelines for biobank governance. This is achieved by drawing attention to the processes through which people embed new knowledge into the stock of knowledge they already possess ^43^. The use of Citizen-Expert panels was also explored, through which an informed discussion is enabled among a heterogeneous sample of citizens—with respect to age, gender, educational background, and experience with medical research. An information session followed by time for clarifications prior to discussion, is facilitated by experts—researchers and professionals with experience and expertise in biology, medicine or ethics, research ethics committee members and lawyers. Subsequently, discussions between citizens and experts, and among citizens following the information session were analysed to inform the governance of biobanks ^43^.

In Australia, Canada and the United States, the general public’s opinions on several of these key factors directly influenced the development of institutional and governmental policies for the establishment of biobanking and genetic research projects—including the BC BioLibrary, Michigan BioTrust and other NIH studies ^11, 13, 15, 28– 30, 34, 35, 47, 63^. Within the United States particularly, engagement and research on stakeholder perceptions contributed towards changes within the regulatory landscape; namely, the enacting of the Genetic Information Non-discrimination Act (GINA) in 2008, the NIH Policy for Genome-Wide Association Studies (GWAS) in 2008 and Genomic Data Sharing in 2015 and with respect to the revisions of the Common Rule which were proposed in 2011 and became effective in 2018 ^13^.

Across African countries, research eliciting communities’ perceptions of biobanking and genetic research has been leveraged to inform the H3Africa consortium’s operating protocol ^26, 59, 65, 66, 68^. Prior to this, local differences in biobanking guidelines and a paucity of studies documenting people’s views on the ELSI of biobanking and genetic research, made international collaboration difficult ^73^. Addressing these concerns effectively was key to establishing the H3Africa initiative successfully ^51^. Engaging with the community also identified a generational shift in people’s motivation to participate. A model of relative solidarity was conceived to take into account the emphasis placed on personal autonomy and self interest by the youths, alongside the communal approach to decision making that was observed in the older community members ^20, 58^.

Drawing from these findings, the Tygerberg Research Ubuntu-Inspired Community Engagement (TRUCE) model provides a framework for community engagement that is “measurable, reliable, and relevant”—particularly for biobanking and genetic research in the African context and other Low and Middle Income Countries (LMICs). The TRUCE model promotes co-creation of engagement strategies, co-ownership of knowledge production, and consultation at each stage of the research process ^20^.

Similarly in New Zealand, ‘He Tangata Kei Tua: Guidelines for Biobanking with Māori’ proposes a relational model for the governance of research involving the Māori. It was developed through consultations with community members and leaders of the Māori ^64^. These projects offer examples for how engagement can be leveraged to develop regulatory guidelines for the conduct of biobanking and genetic research while respecting people’s values and beliefs.

Table 5 summarises the findings with respect to expectations and suggested practices emerging from engaging general and specific publics in biobanking and genetic research.

**Table 5.** Summary of findings from engaging people in biobanking and genetic research.

Region	Country	Date Published	Findings
Africa	Cameroon, Ghana, Kenya, Nigeria, South Africa, Tanzania	2010-2019	1. Evidence of therapeutic misconception 2. Practical relationship-based ethics to support principle-based paradigms 3. Use local knowledge while communicating about research 4. The rules of engagement 5. Myth of anonymity 6. Relative solidarity model: generational shift in the factors influencing participation 7. TRUCE model: participants as co-producers
Asia	Jordan	2019	1. Need for regulations to protect the rights of donors
Australasia or Oceania	Australia, New Zealand	2012-2017	1. Sample contribution as *Taonga*—a treasured possession that is gifted to researchers 2. Community approval for research 3. Biobanks need to act as stewards of samples and associated data
Europe	Austria, Belgium, Croatia, Denmark, Finland, Greece, Netherlands, Norway, The United Kingdom	2013-2018	1. Extensive engagement required to harmonise biobank policies across diverse populations 2. Warrants of trust 3. Shifting from engaging publics to engaging knowledges 4. Evidence of therapeutic misconception
North America	Canada, The United States	2007-2019	1. Parents and guardians to be gatekeepers for children participating in research 2. Need for transparency in the conduct of research 3. People are generally unaware about regulations around biobanking and genetic research 4. Appropriate translations of information sheets and consent forms should be provided to participants 5. Need for more engagement with vulnerable populations 6. Enabling participants to become partners in research 7. Community approval for research and publications in primary and secondary uses of samples and data

### vi) What are the barriers to conducting engagement?

Engaging general and specific publics has been limited by the fact that it is time consuming, costly and difficult to evaluate in terms of its effectiveness ^15^. Public consultations in general, have been criticised “for placating the public and speeding product development, as mechanisms for ‘engineering consent’, as framed by narrow questions” ^30^. In this regard, different engagement strategies employed by biobanks have the potential to legitimise research interests through a process of bio-objectification—where populations are constructed to be imbued with particular attitudes and characteristics ^19^. Minipublics engaged through deliberation run the risk of legitimising interests of elites in the group, since those with reduced agency, may not have the opportunity or social capital to participate equally. Therefore, measures must be taken in the design of the deliberative process to mitigate this issue ^34, 35^. A major challenge experienced in community engagement was developing a clear definition of the ‘community’ being involved; especially in cases where potential participants for biobanking research have very few attributes in common and associations made can be as broad as people visiting the same hospital ^13, 37, 68^. Similarly, research on people’s perceptions of biobanking and genetic research may lead to an inability to discern themes that are relevant across a general population when sample sizes are relatively small or homogenous ^63^.

Challenges also arise from the complexity of topics involved in biobanking and genetic research and the goals of public and community engagement. Low levels of awareness about biobanking and genetic research can make the process of eliciting people’s perceptions on the topics difficult ^15^. In addition to this, language barriers can limit participant’s understanding of the information provided and the effectiveness of engagement ^45^. Sufficient time may not be allocated towards community engagement, which can be construed as ‘tokenism’ and fail to create trusting partnerships between researchers, research institutions and participants ^20^. Apart from these concerns, it has been observed that people’s opinions about their enrolment in a hypothetical study may not reflect their attitudes towards actual participation in a biobanking or genetic research project ^49, 51^.

## Discussion

This literature review of peer reviewed empirical studies on public and community engagement in the context of biobanking and or genetic research shows a predominance of publications in the last decade, spanning 2007 to 2019. We found that people were primarily concerned with facilitating biobanking and genetic research across countries and within communities, and sustaining research over time. The use of participatory approaches helped in identifying ethical concerns emerging from people’s values and beliefs, and in situating governance mechanisms to address ethical challenges in people’s experiences. In addition to the instrumental value of such approaches in enabling and sustaining ethical biobanking and genetic research, there are also core intrinsic values that emerge from participation approaches. These include recognition and respect of sample contributors as ‘partners’ or ‘co-producers’ in new knowledge; trust in non-exploitation and outcomes of public good; and the importance of responsible stewardship approaches in protecting participants’ interests and safeguarding their trust over time. These fundamental cornerstones of best practices that should ground biobanking and genetic research undertakings are relevant to the Indian context as well. This is evident from preliminary work conducted within India, that emphasised the need to protect participants’ rights in the governance of biobanking and genetic research ^6, 7, 9, 74, 75^.

The papers reviewed showed a spectrum of approaches that align with the continuum along which varying degrees of public participation may be elicited. This included approaches which: increased people’s awareness of biobanking and genetic research (communication); elicited general and specific publics’ perceptions on ethical issues in biobanking and genetic research governance (consultation); and finally, engaged general and/or specific publics to actively (and in some instances, collaboratively) incorporate their input within the policymaking and decision making process.

Our findings highlight the nuanced differences between participatory approaches involving ethnolinguistic communities, and specific populations with vulnerabilities, as opposed to the general public and other categories of specific publics. While broad ELSI can be identified by engaging people belonging to the general public, community level interaction is imperative to discern how different groups interpret and resolve these concerns based on their own values and beliefs associated with this research. Engagement helped communities negotiate ethical concerns and acceptable limits in biobanking and genetic research. A case in point is that, at the community level, people expressed their willingness to be more involved in the research process and increase their knowledge about biobanking and genetic research, by making the shift from passive participants to partners in research ^35, 37, 46, 48, 60, 64, 66, 67^. These desires were coupled with the need for culturally appropriate engagement mechanisms which respected people’s customs and beliefs, by establishing the rules of engagement ^38, 55, 65, 67, 68^. This forms a ‘relational approach’, which enables mutually beneficial research ^48^. These considerations have become relevant in the aftermath of the Havasupai Indians’ case, where a settlement was reached owing to the secondary use of their HBM samples in studies that were not approved by the community ^72^. Adopting a participatory approach that gives emphasis to building relationships with communities is relevant to India, given the diversity in populations and the need for ensuring trust that is required to sustain this form of research over time; as well as to mitigate legal issues and the possible stigmatisation of specific communities resulting from biobanking and research ^76^.

At the same time there emerged a need to shift the focus of engagement from merely representing different sections of the public to engaging ‘knowledges’. This offers a means to overcome the issue of bio-objectification—where different populations are imbued with particular attitudes and characteristics ^19^. People are able to identify appropriate limits for biobanking and genetic research by embedding new knowledge they receive from interacting with experts into the stock of knowledge they already possess ^43^. Due to differences in economic and sociocultural factors such as literacy, social groupings, cultural traditions and religious beliefs in India ^7^, we see the need for ‘engaging knowledges’, embedded in wider social and cultural relations.

Moving forward, it is necessary to explore all levels of the participatory spectrum with respect to general and specific publics within the Indian context. Several tools identified in this review can be adopted within the Indian context to improve communication, facilitate consultation and institute engagement towards the governance of biobanking and genetic research ( Table 2). Steps must be taken to enhance people’s awareness about biobanking and genetic research, and elicit general and specific publics’ perceptions of biobanking and genetic research within the country. Similarly, considerations must be made to actively involve the public and other stakeholders in the policymaking process and enable participants to become partners within the conduct of research—via engagement. Here, stakeholder groups such as scientists, regulators, policymakers, patients, legal practitioners, religious group representatives, activists and non-governmental organisations, among several others have previously been identified, as potential influencers in the governance of biobanking and genetic research within the country ^8^. We see that participatory approaches such as public meetings, community durbars, focus group discussions, interviews, deliberations, citizen-expert panels and community advisory boards can be adopted to fulfil these criteria. While public deliberation as an engagement methodology has potential in India, care and effort need to be taken in the design of such processes to ensure diversity in representation, and guarantee equity and fairness in deliberation. Similarly, in the case of focus group discussions and interviews, considerations must be made to enable the otherwise unheard voices.

The above factors are essential milestones in realising the potential of ethical biobanking and genetic research in India through appropriate means. An important lesson for India is that the implementation of engagement and other participatory approaches is resource dependent. This stresses the importance of securing adequate funding and allocating significant amounts of time for the planning for communication, consultation and engagement in biobanking and genetic research ^20^. Furthermore, given the complexity of topics involved in biobanking and genetic research, sufficient effort must be made to make information available to people in easily comprehensible formats, and provide appropriate, culturally relevant translations where necessary.

In this regard, engaging people must not be seen as an obstacle in the conduct of biobanking and genetic research. While money and time may seem to be a luxury that a developing country like India can ill afford, it must be weighed against the greater trust such processes imbue in the public and other stakeholders; and the resultant sustainability and social value of research that are realised over the long term.

Finally, although we analysed a large number of papers from across the globe, our review does have limitations. These include the constraints inherent to the choice of language (i.e. English), keywords used in our searches and the fact that we did not include projects based solely on statistical (non-participatory) methodologies. This could explain the absence of papers which reported on empirical findings from Latin America, Russia and Asia (excluding Jordan), despite the prevalence of biobanking and genetic research undertakings in these regions. Apart from this, since many papers reported on perceptions that were not linked to participation in an ongoing biobanking or genetic research project, there may be some differences in attitudes towards actual participation that may not have been identified in our review.

## Conclusion

As biobanking and genetic research are gaining prominence in India, engaging relevant stakeholders is an ethical imperative and a means to ensure legitimacy within the conduct of research. Participatory approaches to engagement are particularly relevant for the Indian context where awareness about science and health related issues is low and a power dynamic exists between researchers and participants. With respect to the governance of biobanking and genetic research, our results highlight the importance of trust, transparency, gate-keeping, custodianship and using culturally appropriate engagement strategies; particularly when vulnerable populations are involved and if there has been a history of exploitation within the conduct of research. Engagement allowed researchers to understand how people’s values, opinions and experiences relate to the research process; and enabled participants to become partners within the conduct of research. Given the inherent diversity of the Indian population, there is a need for communication, consultation and engagement in relation to biobanking and genetic research. Tools such as public meetings, community durbars, focus group discussions, interviews, deliberations, citizen-expert panels and community advisory boards could be used in this regard. Constructs such as ‘co-production’, ‘engagement of knowledges’, ‘rules of engagement’ and ‘stewardship’ emerge as significant mechanisms that can address the governance of biobanking and genetic research within the country. However, the value of these findings require further sociological research to tailor them for the Indian context.

## Data availability

### Underlying data

All data underlying the results are available as part of the article and no additional source data are required.
