# Supplementary material for: Engaging publics in biobanking and genetic research governance - a literature review towards informing practice in India
Source: Wellcome Open Res. 2021 Feb 16;6:5. Originally published 2021 Jan 13. [Version 2] doi: 10.12688/wellcomeopenres.16558.2 (PMC11026954; doi:10.12688/wellcomeopenres.16558.2)
Supplement: Peer review history

# Reviewer report: Reviewer response for version 2

**DOI:** 10.21956/wellcomeopenres.18352.r74901

**Published:** 2024-04-18

**Authors:** Signe Mežinska

**Affiliations:** 1 University of Latvia, Rainis Boulevard, Latvia

This is a well-written article based on a literature review that synthesizes existing empirical literature to explore public engagement strategies applicable to biobanking and genetic research governance in India. Additionally, the authors aim to understand the significance of public engagement in maintaining trust and support for biobanking and genetic research within the Indian context. The authors are looking for practically applicable approaches to a specific sociocultural context characterised by low awareness of science and health issues, alongside existing unequal power dynamics between researchers and research participants.

 In the second version of the article, the authors have taken into account and integrated the feedback provided by previous reviewers into their work. The discussion and conclusion sections show potential strategies for engaging stakeholders in a manner that is both meaningful and practical.

 For future research, the authors might find it useful to work on the development of a culturally informed model tailored specifically for public engagement in biobanking within India. Such a model, rooted in local cultural values and ethical frameworks, holds the potential to forge stronger connections and foster sustainable practices within the biobanking landscape.

Is the work clearly and accurately presented and does it cite the current literature?

Yes

If applicable, is the statistical analysis and its interpretation appropriate?

Not applicable

Are all the source data underlying the results available to ensure full reproducibility?

No source data required

Is the study design appropriate and is the work technically sound?

Yes

Are the conclusions drawn adequately supported by the results?

Yes

Are sufficient details of methods and analysis provided to allow replication by others?

Yes

Reviewer Expertise:

Empirical bioethics, ethics of biobanking, ethics of biomedical research, medical ethics.

I confirm that I have read this submission and believe that I have an appropriate level of expertise to confirm that it is of an acceptable scientific standard.

# Reviewer report: Reviewer response for version 2

**DOI:** 10.21956/wellcomeopenres.18352.r74907

**Published:** 2024-03-29

**Authors:** Sunita Sheel

**Affiliations:** 1 Health, Ethics and Law Institute for Training Research and Advocacy (HEaL Institute) and Vidhayak Trust, Mumbai, Maharashtra, India

It is a relevant issue and therefore an important contribution, especially since it is aimed at India and in relation to biobanking and allied thematics. 

 Review comments:  

 I have chosen 'partly' to some of the review questions. The comments below may help explain the choice of 

 1. There are range of methods of lit review. Will be important to mention which of these methods used for the lit review which this manuscript presents; and the rationale for choosing this particular lit review method. Later in methods it says 'targeted lit review': Is that a type of literature review method? Please explain it briefly and mention the rationale for choosing this particular lit review methods over the others?

 2. It is not adequately clear as how a lit review of body of work from across the world in this specific space enable extrapolating insights into the existing scholarship to the biobanking spaces in the Indian context. Power dynamics, asymmetrical power relationships between researchers and study communities, diversity across section of population in a specific country, and multitude of ethnic communities, minoritized communities across range of axes is fairly ubiquitous  regardless of. If so, it will benefit and strengthen the manuscript as what is specific about India that these insights/results could be applicable to; and the methodological approach adopted to undertake this targeted lit review. 

 For exampled, P no 4 it says, 'We focused ....; and an unequal power dynamic exists between the researchers and research participants7. ...."  This applies to many other contexts. 

 3. P.no 3 | Opening para enlists questions being explored through this lit review.  

 It appears that an important questions alongside these ones in line with the purpose of undertaking such a lit review needs to be articulated here clearly and explicitly. For example, Which of these insights could inform public engagement in biobanking spaces in India? Why? How? 

 Responses to such a question would then shape recommendations for peer community in India in biobanking spaces.

 P no 4: It mentions the two data bases searched to retrieve relevant papers/materials for review. 

 Will be helpful to explain the rationale for choosing these two amongst others.

 P no 4 | Sub-section 'Analysis': It mention framework method used and how coding was done related matters. 

 Will be helpful for the peer community engaged with this specific theme and allied areas/thematics to know the outcomes of the analysis in the form of codes; and mid-stream analysis outcome by sharing codes developed, and those that emerged through iterative thematics analysis within the Framework Method approach. It will help strengthen the piece, and also provide an opportunity for the peer community. It could be provided as a supplementary materials. It will be immensely helpful. 

 P no 7 | A minor observation: It says 'Wellcome'. Maybe it should be Wellcome Trust. 

 Responding to some of these comments/questions will lead to revisit 'conclusion' and also some revisions to the Methods, Results' and discussion sections.

Is the work clearly and accurately presented and does it cite the current literature?

Yes

If applicable, is the statistical analysis and its interpretation appropriate?

Not applicable

Are all the source data underlying the results available to ensure full reproducibility?

Partly

Is the study design appropriate and is the work technically sound?

Partly

Are the conclusions drawn adequately supported by the results?

Partly

Are sufficient details of methods and analysis provided to allow replication by others?

Partly

Reviewer Expertise:

Women and Health; Health Policy, Bioethics-Health-Human Rights, Community Engagement and allied thematics.

I confirm that I have read this submission and believe that I have an appropriate level of expertise to confirm that it is of an acceptable scientific standard, however I have significant reservations, as outlined above.

# Reviewer report: Reviewer response for version 2

**DOI:** 10.21956/wellcomeopenres.18352.r42650

**Published:** 2021-02-23

**Authors:** Kieran C. O'Doherty

**Affiliations:** 1 Department of Psychology, University of Guelph, Guelph, ON, Canada

In my previous review, I noted that this manuscript made a strong contribution and I only made minor suggestions for improvement. The authors have addressed these issues very well and I have no further comments on this revised version of the article. My congratulations to the authors on a well-executed review and for using it to develop important insights for the Indian context.

Is the work clearly and accurately presented and does it cite the current literature?

Yes

If applicable, is the statistical analysis and its interpretation appropriate?

Not applicable

Are all the source data underlying the results available to ensure full reproducibility?

No source data required

Is the study design appropriate and is the work technically sound?

Yes

Are the conclusions drawn adequately supported by the results?

Partly

Are sufficient details of methods and analysis provided to allow replication by others?

Yes

Reviewer Expertise:

Public deliberation; public engagement; social psychology; bioethics; ELSI; qualitative methods

I confirm that I have read this submission and believe that I have an appropriate level of expertise to confirm that it is of an acceptable scientific standard.

# Response

**Published:** 2021-02-24

**Authors:** Manjulika Vaz

Dear Prof O Doherty,

 Thank you so much for the valuable comments and suggestions and overall feedback on our paper. You were one of the leading authors we referred to constantly through this and earlier work on public deliberation and biobanking.

 It's an honor and a privilege to have been reviewed by you.

 With kind regards,

 Manjulika, Prasanna and team

# Reviewer report: Reviewer response for version 1

**DOI:** 10.21956/wellcomeopenres.18245.r42141

**Published:** 2021-02-03

**Authors:** Kieran C. O'Doherty

**Affiliations:** 1 Department of Psychology, University of Guelph, Guelph, ON, Canada

This article reports on an extensive literature review of studies on engaging publics in biobanking and genetic research. The purpose of the review is to inform practice in India. Overall, this is a very well written article. It addresses an important issue and seeks to synthesize international research for local application. The review provides a high level overview of available research, while nevertheless providing nuance and clear conceptual guidance. I feel that the article makes an important contribution that is worth publishing and I have only minor suggestions for improvement.

1. The article uses the term “non-positivist methods” and “positivist approaches” (p.3). While I have a sense of what the authors are referring to and this use of the term is not unusual, it is imprecise. In particular, there are various groups that have been identified by historians of science that have associated with the idea of “positivism”. However, these groups had important differences in how positivism was conceived. More importantly, it is unlikely that the 133 publications identified by the authors as reporting on “positivist approaches” are in fact linked by a clearly articulated epistemology that can unambiguously be called “positivist”. In short, using the term “positivist” to denote a common characteristic underlying a wide variety of contemporary studies is unclear (see Thomas Teo and Hank Stam who have written about this issue in detail). Rather than use the term “positivism” the authors should instead provide precise and unambiguous criteria for what led to the rejection of particular studies in the review (for example, rejecting studies that only reported statistics, which is also mentioned on p.3 is a more precise criterion). Similar problems are associated with the term “non-positivist methods” used earlier on p.3.
2. Research question vi) “What are the barriers to employing engagement?” is a little vague (since it begs the question 'employed to what ends?). Perhaps “What are the barriers to conducting engagement?” (or similar) would be more precise?
3. The authors state that “… was undertaken manually by one author (PW), then reviewed and verified by another (MV).” (p.3) Two of the authors have the initials MV, so further specificity is required.
4. “Participants at the public level were referred to as citizens or constituted a minipublic—where the group of citizens being engaged were seen to be a representative sample of the larger population.”(p.5) This statement is not correct for many of the studies cited following the statement. In particular, the way in which minipublics could be seen as representative (or not) of the larger population is acknowledged to be complicated. To be statistically representative of a larger population, a minipublic would typically need several hundred participants (depending, of course, on the size of the population to be represented). To be politically representative, a minipublic would have to be the result of some kind of electoral or other political process. Neither of these conditions is the case for most of the studies cited, and most of them do not claim that the minipublics are representative of larger populations in a straightforward way. These issues are discussed in more detail by Longstaff & Burgess (2010) ^1^ and Steel *et al.* (2018). ^2^
5. The authors make a useful distinction between engaging specific publics and general publics. However, there is some additional nuance here. For example, the deliberative process reported by O’Doherty *et al.* (2012) engaged “general publics” but members of specific publics were invited to inform the participants in the deliberation about the views of their community, and so ensured that these views were taken into account in the deliberation. This is a feature that is common to several other deliberative processes as well.
6. The authors discuss the barriers that might be associated to engagement (p.9). Overall, this section does a good job of characterizing concerns and barriers. However, some of the references used to support the points of the authors are misrepresented. For example, it is correct that “Minipublics engaged through deliberation have been criticised for legitimising the interests of elites in the group, since lay publics and underrepresented groups may not have the opportunity or social capital to participate equally.” However, the two references provided to support this claim (Molster *et al. *2012; O’Doherty *et al.*, 2012) don’t conclude this from their studies. Rather, they note it as a problem that should be recognised, but that can be addressed with good design and implementation of deliberative processes. Indeed, both of those studies are evidence that deliberative processes can help to mitigate these kinds of problems. This issue comes up again in the Discussion section on p.11. The authors state that “However, conducting public deliberations may prove to be difficult in India, as it poses the risk of legitimising elite interests in the group, since underrepresented groups may not have the opportunity or social capital to participate equally.” This is indeed a risk that must be acknowledged. However, public deliberation should not be rejected on the grounds of this reasoning. The studies cited in this review that have conducted on public deliberation on biobanks constitute evidence that public deliberation can be conducted in such a way that mitigates many asymmetries in social standing and power across society. The argument that deliberation can legitimise elite interests may be supported in some instances, but many more instances have shown that public deliberation is a powerful tool to specifically open up important policy questions to non-elite stakeholders and broader publics.
7. Following on from the previous point, the authors state that “Therefore, particularly for the Indian context, adequate considerations should be made to incorporate views across a diverse group of people.” (p.11). This is precisely the goal of public deliberations and the studies cited in the review demonstrate that this can be achieved.

Is the work clearly and accurately presented and does it cite the current literature?

Yes

If applicable, is the statistical analysis and its interpretation appropriate?

Not applicable

Are all the source data underlying the results available to ensure full reproducibility?

No source data required

Is the study design appropriate and is the work technically sound?

Yes

Are the conclusions drawn adequately supported by the results?

Partly

Are sufficient details of methods and analysis provided to allow replication by others?

Yes

Reviewer Expertise:

Public deliberation; public engagement; social psychology; bioethics; ELSI; qualitative methods

I confirm that I have read this submission and believe that I have an appropriate level of expertise to confirm that it is of an acceptable scientific standard, however I have significant reservations, as outlined above.

## References

[1] : Recruiting for representation in public deliberation on the ethics of biobanks. *Public Underst Sci* .2010;19(2) : 10.1177/0963662508097626 212-24 10.1177/0963662508097626 20533799

[2] : Multiple diversity concepts and their ethical-epistemic implications. *Eur J Philos Sci* .2018;8(3) : 10.1007/s13194-018-0209-5 761-780 10.1007/s13194-018-0209-5 30956737 PMC6414089
